# Indicator species of former lead (Pb) ore mining areas

**DOI:** 10.1007/s11356-024-35579-7

**Published:** 2024-11-26

**Authors:** Monika Podgórska

**Affiliations:** Institute of Biology, Jan Kochanowski University, Uniwersytecka 7, 25-406 Kielce, Poland

**Keywords:** Bioindicators, Deciduous forest species, Lead post-mining mounds, Vascular plants

## Abstract

The main objective of this study was to determine whether species that are considered to be indicator species for former iron ore mining areas also have value as indicators for remnants of former lead ore mining areas. The study was conducted at an abandoned post-mining field dating from the sixteenth century with visible remains from the exploitation of lead ore deposits (post-mining mounds, PMM). In each of the 41 plots (21 on PMM and 21 in the surroundings of PMM), an inventory was conducted of all vascular plants growing in the vegetation layer indicating both their coverage in percent and analyzing them based on indicator species for former iron mining sites. Additionally, soil samples were taken from each, and chemical analyses were done: pH in H_2_O; contents of Ca, and Pb; and available forms of K, P, and Mg. Changes in the chemical properties of the soil impacted the diversity of the flora of the analyzed area. In the 21 research plots established on the PMM of the former lead ore mining area, as many as 18 species of mesophilic deciduous forest considered to be indicator species for former iron mining areas were identified. The analyses conducted indicate a strong preference for these species for the soil occurring on the lead PMM. Indicator species for former iron mining areas can also be a good indicator for former lead ore mining areas.

## Introduction

Indicator species (bioindicators) are used to determine the state of another species or entire ecological system. In making this determination, both biotic and abiotic parameters must be taken into account, as well as anthropogenic impact (Holt and Miller 2010; Manickavasagam et al. 2019). Bioindicators can be single organisms, populations, multispecies biocenoses, ecosystems, and even landscapes. When bioindicators are individual organisms, their presence, absence, or characteristic population numbers provide information about the features and properties of a given area (Al-Maliki et al. 2021; Di Fiore et al. 2022; Ramírez et al. 2021; Subramanian 2023).

Issues associated with the indicator role of plants have been known for quite some time. Such issues concern, among other things, their role as indicators of habitat conditions in the general sense—these are the so-called Ecological Indicator Values developed for Central Europe (Ellenberg et al. 1991) or for Poland (Zarzycki et al. 2002). Other works have discussed the indicator role of selected groups of species, indicator species for ancient woodlands (Hermy et al. 1999; Dzwonko and Loster 2001; Hemmings 2022), or indicators for plant communities (Lindenmayer et al. 2000; Roo-Zielińska 2014; Xu et al. 2023), as well as landscape indicators (Ares et al. 2001; Dyderski et al. 2017; Fry et al. 2009; Pitchford et al. 2000).

A separate issue is related to the indicator role of plants in post-mining areas. Despite the considerable interest of researchers in these types of habitats, there are few works that deal with the usage of plants as bioindicators for post-mining sites. There are certain propositions for the use of vascular plants to diagnose post-mining soil substrate using ecological indicator values (Becker and Dierschke 2008; Pietrzykowski and Krzaklewski 2006), and also suggestions for the use of defined species of plants for the revegetation of post-mining areas (Festin et al. 2019; Pająk et al. 2018). One group of works also focuses on the indicator role of metal-tolerant plants in these areas (Adlassnig et al. 2016; Babst-Kostecka et al. 2016; Baker et al. 2010; Conesa et al. 2007). However, none of these researchers developed a list of indicator species for post-mining sites. It is a laborious task because such a list of indicator species should be based on floristic and phytosociological analysis and comparison with soil analyses. This type of work was done by Podgórska (2019) in order to determine the group of indicator species for former iron ore mining areas.

In general, “the former ore mining” involved the exploitation of deposits using primitive methods in the area of the Old-Polish Industrial Region (southern Poland, Central Europe)—the oldest and, until the late nineteenth century, the largest mining and metallurgy region in Europe (Guldon 2001; Orzechowski and Suliga 2006). The beginnings of ore extraction in this region date back to the twelfth to fifteenth centuries, that is, from the earliest stages of the development of blast furnaces. The last mines shut down operations around the mid-twentieth century (Krajewski 1947; Podgórska 2019). The most characteristic feature of this ancient mining practice was the manual extraction of ore (mainly iron ore, less frequently lead ore), involving the excavation of many narrow (0.5–1.5 m width), fairly deep (from several to several dozen meters depth) shafts, which in a given mining field were dug quite close to one another (Pierściński 2001; Podgórska 2010). The ore was separated from the excavated material and brought up to the surface, and the unwanted and completely unprocessed remaining material was left around the shaft heads. Nowadays each mining field comprises many post-mining mounds built up of remaining material extracted from deeper rock layers (from the ore-bearing horizon). Around the post-mining mounds, in the untransformed areas, there are formations of the layer above the ore-bearing horizon.

The result of a 10-year study on the flora, plant communities, and soil environment (Podgórska 2019) conducted on the remnants of former iron ore mining (PMM) within 50 former mining fields (150 permanent research plots on PMM and 150 in the area surrounding the PMM) distributed over an area of 700 km^2^ within the bounds of the Old-Polish Industrial Region was the discovery of a group of indicator species for these habitats (Table 1). These indicator species let us find former iron ore mining areas even when PMM is no longer visible in the terrain (without the need to conduct costly archaeological research).

**Table 1:** List of indicator species for the former iron ore mining areas (Fe post-mining mounds) according to Podgórska (2019)

No	Name of species	No	Name of species
1	*Viola reichenbachiana*	16	*Campanula persicifolia*
2	*Sanicula europaea*	17	*Lathyrus vernus*
3	*Melica nutans*	18	*Paris quadrifolia*
4	*Carex digitata*	19	*Pulmonaria obscura*
5	*Daphne mezereum*	20	*Brachypodium sylvaticum*
6	*Hepatica nobilis*	21	*Milium effusum*
7	*Festuca gigantea*	22	*Platanthera chlorantha*
8	*Actaea spicata*	23	*Phyteuma spicatum*
9	*Galeobdolon luteum*	24	*Stellaria holostea*
10	*Stachys sylvatica*	25	*Astrantia major*
11	*Carex sylvatica*	26	*Neottia nidus-avis*
12	*Asarum europaeum*	27	*Galium odoratum*
13	*Polygonatum multiflorum*	28	*Melittis melissophyllum*
14	*Galium schultesii*	29	*Lilium martagon*
15	*Poa nemoralis*	30	*Aruncus sylvestris*

This significant finding led the author to hypothesize that the indicator species for former iron ore mining sites may also indicate former lead ore mining sites (because of the similar way and time of exploitation and similar morphology and origin of post-mining mounds). Therefore, the main objective of this paper was to determine whether indicator species for former iron ore mining areas developed by Podgórska (2019) also have indicator values for remnants of former lead ore mining areas.

## Methodology

### Study area

**Fig. 1:**
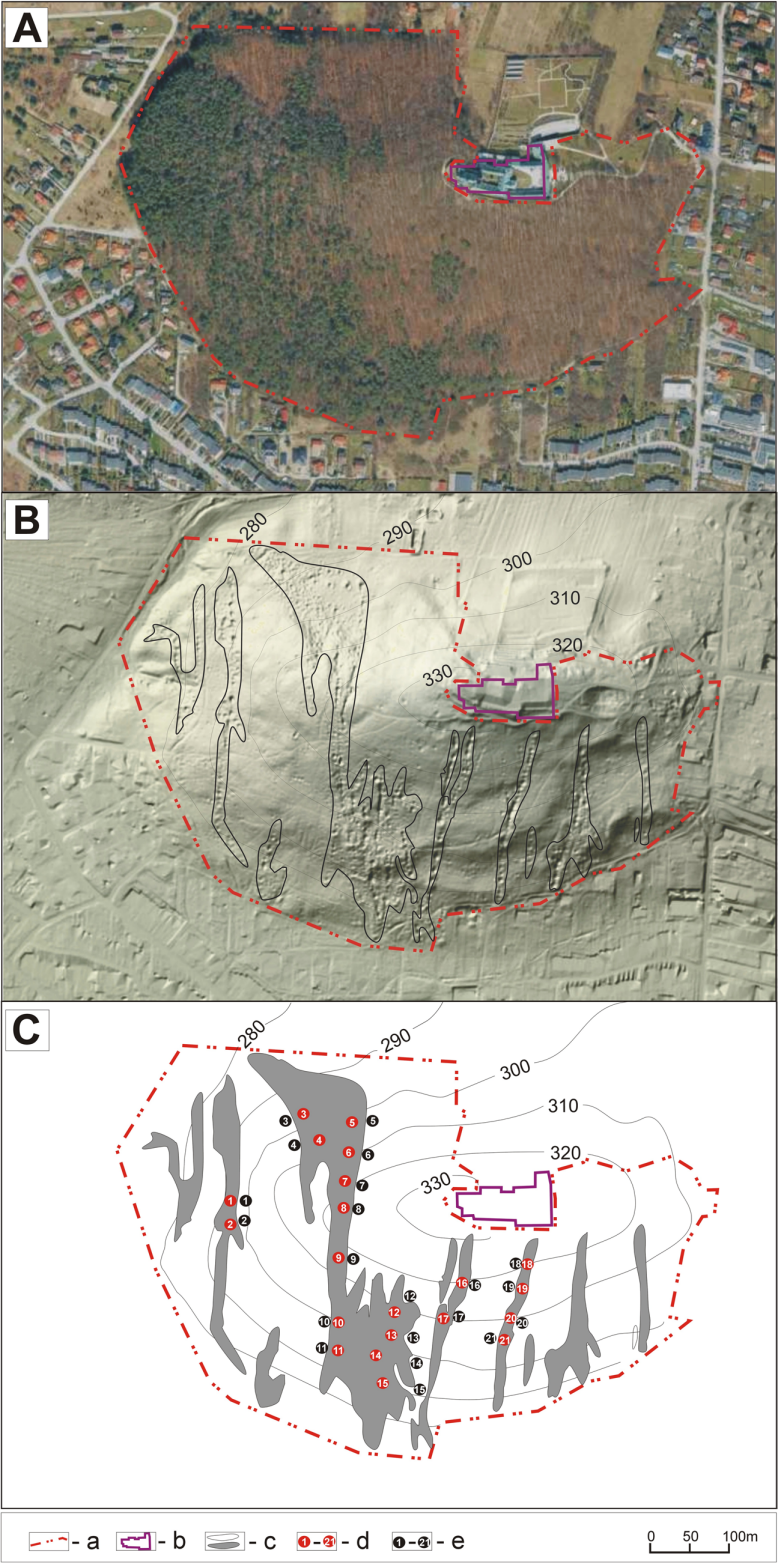
Study area—the Karczówka Nature Reserve: **A** satellite image, **B** LIDAR image with visible Pb post-mining mounds (PMM), and **C** scheme of distribution of 41 research plots (a, boundary of the reserve; b, monastery; c, complexes of PMM; d, research plots on lead PMM; e, research plots in the surroundings of lead PMM)

The study area comprised the Karczówka Nature Reserve, which was established in 1953 on an area of 26.62 ha. Karczówka is a scenic landscape reserve, partially located within the Świętokrzyskie Mountains (southern Poland, Central Europe). The aim of the creation of the reserve was to preserve a fragment of a nearly 200-year-old woodland forming the scenically beautiful surroundings of a historic sixteenth-century monastery for social and cultural reasons (Fig. 1A). The reserve includes the peak and slopes of Karczówka Hill, formed of Middle and Upper Devonian limestone, and is overgrown by forests. The slopes of Karczówka are formed of coarse-grained limestone on which numerous surface karst formations can be seen (Gałuszka et al. 2018). Near the peak, there are grey rocky and plate limestones. Apart from its scenic value, the reserve is also valuable in geological terms. In the past (from the sixteenth to the nineteenth centuries), the steep slopes of the hill were an important center of lead ore extraction for the needs of the Old-Polish Industrial Region (Pierściński 2001). Numerous remnants of the historic mining operations have survived to the present day: craters and depressions in the area of the shaft heads, mining trenches, and PMM, currently overgrown with forest and absorbed into the surrounding landscape.

### Study design

The research was conducted in the years 2021–2023 (in the vegetative season, from April to October). It covered a fragment of a former mining field located on Karczówka Hill (50° 52′ 02″ N, 20° 35′ 13″E) with visible remains of the former exploitation of lead ore mining (Fig. 1B). Among the former PMM, 21 research plots were set (10 × 10 m each). For comparison, 21 plots of 10 × 10 m were also set on the untransformed land surrounding the PMM (at a distance of 20 m from each mound). In total, 42 research plots were set (Fig. 1C). In each of these, an inventory was conducted of all vascular plants growing in the vegetative layer (herbaceous plants and seedlings of trees and bushes), determining at the same time their cover in percent on each of the research plots using a modified scale of degrees of abundance and cover according to Braun–Blanquet (Dzwonko 2007), where + = 5%, 1 = 10%, 2 = 20%, 3 = 37.5%, 4 = 62.5%, and 5 = 87.5%. In addition, a soil sample was taken from the center of each plot at a depth of 5–30 cm using a soil sample auger. In total, 42 soil samples were taken (21 from the former PMM and 21 from the areas untransformed by former lead ore mining activity). Soil samples were collected with a polyethylene scoop and stored in plastic bags.

### Sample and data analysis

All recorded species growing on the soil surface of the PMM and in the surrounding areas (Table 2) were analyzed based on indicator species for former iron ore mining areas (Table 1).

**Table 2:** List of vascular plant species that occur in the former lead ore mining area on the post-mining mounds (PMM) and in the surroundings of PMM in the Karczówka Nature Reserve

Type of habitat			Former Pb post-mining mounds		Surroundings of former Pb post-mining mounds	
Mean total plant cover of 21 plots			60%		15%	
Total number of species			61		35	
No		Name of species	Number of localities	Cc	Number of localities	Cc
1		*Abies alba*	2	48	-	-
2		*Acer platanoides*	11	476	13	309
3		*Acer pseudoplatanus*	13	405	5	119
4		*Aegopodium podagraria*	10	476	17	357
5		*Aesculus hippocastanum*	2	48	1	24
6		*Ajuga reptans*	5	119	-	-
7		*Anemone nemorosa*	3	369	-	-
8		*Anthriscus sylvestris*	-	-	3	71
9		*Asarum europaeum*	21	1819	-	-
10		*Ballota nigra*	-	-	1	24
11		*Brachypodium sylvaticum*	1	24	-	-
12		*Calamntha vulgaris*	1	24	2	48
13		*Campanula trachelium*	2	72	1	24
14		*Carex contigua*	5	119	7	167
15		*Carex digitata*	17	524	-	-
16		*Cerasus avium*	1	24	-	-
17		*Chaerophyllum aromaticum*	-	-	10	238
18		*Clematis vitalba*	5	167	19	452
19		*Convallaria majalis*	2	48	5	142
20		*Cornus sanguinea*	3	71	1	24
21		*Corylus avellana*	1	24	-	-
22		*Cruciata glabra*	5	143	-	-
23		*Daphne mezereum*	2	48	-	-
24		*Dryopteris filix-mas*	3	71	-	-
25		*Euphorbia amygdaloides*	7	190	-	-
26		*Euonymus verrucosa*	5	143	-	-
27		*Fagus sylvatica*	7	190	8	190
28		*Fragaria vesca*	3	95	4	95
29		*Fraxinus excelsior*	3	71	-	-
30		*Galeobdolon luteum*	16	1190	-	-
31		*Galium aparine*	-	-	1	24
32		*Galium odoratum*	1	24	-	-
33		*Galium schultesii*	1	24	-	-
34		*Geranium robertianum*	6	309	6	143
35		*Geum urbanum*	6	143	3	71
36		*Glechoma hederacea*	-	-	2	48
37	*Hedera helix*		2	48	2	48
38	*Hepatica nobilis*		20	1857	-	-
39	*Heracleum sphondylium*		1	24	1	24
40	*Hieracium murorum*		1	24	5	119
41	*Impatiens parviflora*		5	119	10	286
42	*Lamium album*		-	-	1	24
43	*Lathyrus vernus*		2	48	-	-
44	*Lilium martagon*		5	119	-	-
45	*Lonicera xylosteum*		3	71	-	-
46	*Luzula pilosa*		1	24	5	119
47	*Maianthemum bifolium*		11	381	19	667
48	*Melica nutans*		8	238	-	-
49	*Melittis melissophyllum*		2	48	-	-
50	*Milium effusum*		1	24	-	-
51	*Oxalis acetosella*		10	524	18	619
52	*Partenocissus inserta*		1	24	3	71
53	*Poa nemoralis*		1	24	-	-
54	*Pulmonaria obscura*		11	381	-	-
55	*Rubus saxatilis*		3	71	-	-
56	*Sanicula europaea*		10	524	-	-
57	*Sorbus aucuparia*		5	119	1	24
58	*Stachys sylvatica*		2	48	-	-
59	*Stellaria media*		-	-	2	48
60	*Taraxacum officinale*		1	24	1	24
61	*Tilia cordata*		3	71	-	-
62	*Ulmus glabra*		10	286	6	143
63	*Urtica dioica*		1	24	1	24
64	*Veronica chamaedrys*		2	48	2	48
65	*Veronica officinalis*		1	24	2	48
66	*Viburnum opulus*		3	71	-	-
67	*Viola reichenbachiana*		9	249	-	-
68	*Viola mirabilis*		6	167	-	-

*Cc* cover coefficient

The analysis was based on (1) the qualitative share of the indicator species—the number of indicator species identified in each of the 42 studied plots and (2) the quantitative share of indicator species based on the cover coefficient calculated using a modified Braun–Blanquet formula (Dzwonko 2007) as follows:

$$Cover\;coefficient=total\;of\;species\;mean\;cover/number\;of\;permanent\;plots\times 100.$$

The taxonomic nomenclature was based on that developed by Mirek et al. (2020).

The 42 soil samples were air-dried and passed through a 2-mm plastic sieve to remove gravel and rocks, put in plastic bags, and then sent to the Chemical-Agricultural Station located in Kielce (Poland) for analysis. In all of the soil samples, the following chemical analysis was conducted: (1) pH H_2_O using potentiometric method, (2) Ca content (in mg/l) using flame photometry method, (3) content of absorbable forms of K, P, and Mg (in mg/100 g) using Egner–Riehm and Schachtschabel methods, and (4) Pb content (in mg/kg) using the flame atomic absorption spectrometry (FAAS).

The normal distribution of data was checked using the Kolmogorov–Smirnov test, while homogeneity of variance was tested using the Levene test at the significance level of *p* < 0*.*05. As the values in some groups were not consistent with normal distribution and the variance was not homogeneous, the analyses (differences in the analyzed soil traits and in the share of indicator species between the former lead ore mining areas and the untransformed areas) were evaluated using the non-parametric Mann–Whitney *U* test (*P* ≤ 0.05). To illustrate the relationship between the individual chemical properties of the soil samples and the quantitative share (cover coefficients) of individual indicator species in the studied plots, a correlation was calculated using the Spearman correlation coefficient. All statistical analyses were performed using Statistica 6.1 (StatSoft 2003).

## Results

In the 21 research plots established on the former lead ore PMM, 61 species of vascular plants were identified (Table 2). Analyses demonstrated that 18 of them were species of mesophilic deciduous forest considered to be indicator species for former iron ore mining areas (Tables 1 and 3).

**Table 3:** Share of indicator species for the former iron ore mining areas—Fe post-mining mounds (according to Podgórska 2019) and the former lead ore mining area—Pb post-mining mounds

No	Name of species	Pb				Fe
		S (21 plots)	PMM (21 plots)			PMM (150 plots)
		Number of localities	Number of localities	Cc	% of occurence	% of occurence
1	*Asarum europaeum*	0	21	1821	100	46
2	*Hepatica nobilis*	0	20	1857	95	60
3	*Carex digitata*	0	17	524	81	80
4	*Galeobdolon luteum*	0	16	1191	76	56
5	*Pulmonaria obscura*	0	11	381	52	32
6	*Sanicula europaea*	0	10	524	48	92
7	*Viola reichenbachiana*	0	9	429	43	94
8	*Melica nutans*	0	8	238	33	86
9	*Lilium martagon*	0	5	119	24	16
10	*Lathyrus vernus*	0	2	48	10	36
11	*Melittis melissophyllum*	0	2	48	10	18
12	*Daphne mezereum*	0	2	48	10	66
13	*Stachys sylvatica*	0	2	48	10	52
14	*Brachypodium sylvaticum*	0	1	24	5	32
15	*Galium odoratum*	0	1	24	5	18
16	*Milium effusum*	0	1	24	5	28
17	*Galium schultesii*	0	1	24	5	42
18	*Poa nemoralis*	0	1	24	5	38

*Cc* cover coefficient, *PMM* post-mining mounds, *S* surroundings

Of these, on the former lead ore PMM the largest number of stations have *Asarum europaeum*, *Hepatica nobilis*, *Carex digitata*, *Galeobdolon luteum*, *Pulmonaria obscura*, and *Sanicula europaea*. These species also demonstrated high cover coefficients. They comprised the dominant species in the studied plots (Table 2). Significantly, none of the 18 indicator species was identified in the untransformed areas surrounding the PMM (Tables 2 and 3); all of them grew exclusively within the plots established on the PMM, indicating their strong preference for the soil of the former lead ore mining areas. The difference in the share of indicator species between the former lead PMM and the untransformed areas is statistically significant at *P* < 0.00001.

**Fig. 2:**
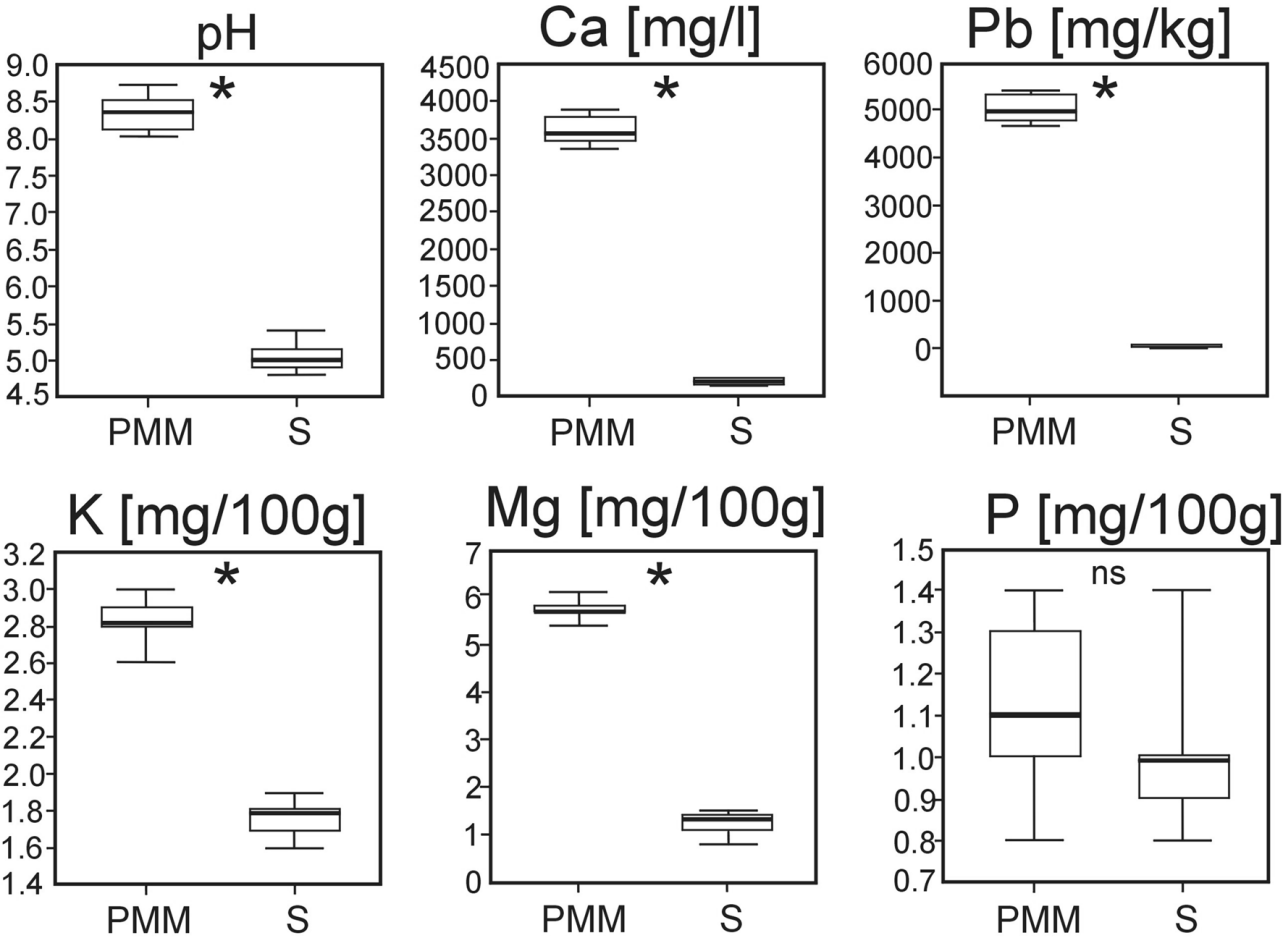
Differentiation of the two habitats on the former lead ore mining area (PMM, post-mining mounds; S, surroundings of the PMM) studied in relation to the 6 properties of soil. White boxes show 25–75% interquartile ranges of values; the horizontal black lines in boxes show the medians; asterisks “*” mean that the differences between plant communities are significant at *p* < 0.05, “ns” mean that the differences between plant communities are not significant at *p* < 0.05

Analysis of the chemical properties of the soil indicated a significant difference between the former lead ore PMM and their surroundings (Fig. 2). The measured soil pH in the former lead PMM was within the range of 8.0 to 8.7 (mean 8.3). In contrast, the soil pH of the untransformed areas was within the range of 5.4 to 4.8 (mean 5.1). On the PMM, a nearly twofold higher Ca content was determined in comparison with the untransformed areas—the mean Ca content in the soil of the former PMM was 3605 mg/100 g, while the mean content in the untransformed areas was 186 mg/100 g. Statistically significant differences between the former lead ore PMM and their surroundings were also identified in terms of available K and Mg (Fig. 2).

The correlations developed between the analyzed individual chemical soil properties of all studied plots (former lead ore PMM and their surroundings) and the cover coefficients of the individual indicator species indicate a strong preference of these species for the soil occurring on the PMM. The share of the 13 species is positively significantly correlated with the chemical soil properties of the former lead ore post-mining sites (Table 4). Among the species most strongly preferring the former lead PMM were *Asarum europaeum* and *Hepatica nobilis* because their share demonstrates the highest correlation coefficients with the highest *P*-values with almost all studied soil properties—pH value, contents of available forms of calcium, potassium, magnesium, and also contents of lead (Table 4).

**Table 4:** Spearman’s correlation coefficient for the quantitative contribution (cover coefficients) of the indicator species of the former Pb post-mining mounds and some selected characteristics of soils

Variables	pH	Ca	P	K	Mg	Pb
*Asarum europaeum*	0.84***	0.84***	0.42*	0.85***	0.85***	0.84***
*Hepatica nobilis*	0.81***	0.80***	0.40*	0.82***	0.81***	0.80***
*Carex digitata*	0.66**	0.67**	0.27^ ns^	0.71***	0.66**	0.67**
*Galeobdolon luteum*	0.71***	0.69***	0.37*	0.73***	0.71***	0.71***
*Pulmonaria obscura*	0.56**	0.57**	0.29^ ns^	0.56**	0.56**	0.57**
*Sanicula europaea*	0.71***	0.70***	0.62**	0.69***	0.70***	0.71***
*Viola reichenbachiana*	0.67**	0.65**	0.60**	0.64**	0.66**	0.67**
*Melica nutans*	0.65**	0,64**	0.62**	0.65**	0.64**	0.65**
*Lilium martagon*	0.56**	0.56**	0.54**	0.55**	0.56**	0.56**
*Lathyrus vernus*	0.37*	0.35*	0.37*	0.37*	0.37*	0.37*
*Melittis melissophyllum*	0.37*	0.35*	0.37*	0.37*	0.37*	0.37*
*Daphne mezereum*	0.37*	0.35*	0.37*	0.36*	0.37*	0.37*
*Stachys sylvatica*	0.37*	0.35*	0.36*	0.36*	0.37*	0.37*
*Brachypodium sylvaticum*	0.26^ ns^	0.23^ ns^	0.26^ ns^	0.25^ ns^	0.25^ ns^	0.26^ ns^
*Galium odoratum*	0.26^ ns^	0.24^ ns^	0.25^ ns^	0.26^ ns^	0.25^ ns^	0.26^ ns^
*Milium effusum*	0.25^ ns^	0.25^ ns^	0.25^ ns^	0.25^ ns^	0.24^ ns^	0.25^ ns^
*Galium schultesii*	0.26^ ns^	0.24^ ns^	0.23^ ns^	0.25^ ns^	0.23^ ns^	0.26^ ns^
*Poa nemoralis*	0.26^ ns^	0.23^ ns^	0.26^ ns^	0.25^ ns^	0.25^ ns^	0.25^ ns^

^*^0.05 > *P* ≥ 0.001; **0.001 > *P* ≥ 0.0001; ***P < 0.0001

*ns* not significant at *p* < 0.05

## Discussion

### Relationship indicator species—soil quality

The study conducted proves that indicator species for former iron ore mining areas (Podgórska 2019) can also be a good indicator for remnants of former lead ore mining areas—from among 30 indicator species for the iron PMM, up to 18 have an indicative value for the lead PMM. The indicator species with the strongest preference for the former lead ore PMM are *Asarum europaeum* and *Hepatica nobilis*, which to a certain degree distinguishes the lead ore PMM from the iron ore PMM, for which the most sensitive indicator species is *Sanicula europaea* (Podgórska 2019). Within the 42 research plots established on a former lead ore mining field in the Karczówka Nature Reserve, 18 indicator species grew exclusively within 21 plots of the former PMM, while not occurring even once in the 21 plots established outside of the area of the PMM yet still in the near vicinity. As chemical soil analysis of the PMM and the untransformed areas (in the vicinity of the mounds) showed, the two types of areas are starkly different habitats. Just as in the case of former iron ore post-mining sites (Podgórska and Jóźwiak 2023), the preference of these species for the former lead ore PMM was associated with the properties of the soil that formed the mounds. These 18 indicator species are species of mesophilic deciduous forest, and they cannot thrive outside of the fertile soil of the lead ore PMM (with higher pH value and contents of calcium, potassium, and magnesium), as the habitats surrounding them are substantially poorer, as the presented research shows. As a result of exploitation during mining operations, originally acidic, sandy soil deposits were covered with material extracted from deeper rock layers (Adamczyk 1965; Podgórska and Jóźwiak 2020), creating the former PMM which comprise considerably richer habitats, than those that can be found in the surroundings. This is an unusual phenomenon as the majority of different “spoil heaps” and various types of works to be found in post-mining areas are characterized by substantially poorer habitat conditions than untransformed areas in their vicinity (Kompała-Bąba et al. 2019; Tambunan et al. 2017). It is also worth noting that the fertile substrate on the lead PMM in the Karczówka Nature Reserve affects the general species richness because, within the 21 plots established on the former lead ore PMM, there is a nearly twofold greater number of vascular plants than that in their surroundings. On the PMM, the core of the flora comprises indicator species for post-mining areas that do not occur in their surroundings. A similar situation can be observed in former iron ore post-mining sites, where species richness was higher than in the surrounding areas (Podgórska 2015) and caused by the increase in the numbers of native species correlated with the soil properties (Strzeleczek et al. 2017)—this is an uncommon phenomenon in comparison with current post-mining areas, where species richness is caused by non-native species (Kabrna et al. 2014; Lemke et al. 2012).

### Application of indicator species for the former post-mining sites

While these indicator species were determined based on research conducted in the foreland of the Świętokrzyskie Mountains, their application as indicators in former ore mining areas may be considerably broader. However, in order for them to be put into broad application, several conditions must be met. A key role is played by the age of the post-mining remnants. This allows for the establishment of plant communities whose floristic composition is not accidental, but rather appropriate for the habitat parameters of the PMM which have formed as a result of mining operations. As many as 90% of indicator species are so-called ancient woodland species (Dzwonko and Loster 2001; Hemmings 2022; Hermy et al. 1999; Palo et al. 2013; Podgórska 2018; Stefańska-Krzaczek et al. 2016; Swallow et al. 2020) whose dispersal is slow, and regeneration of which is possible only in the vicinity of ancient woodlands. Thus, these species will be able to be used as indicators for post-mining areas of Central Europe, which are relatively old. The second condition necessary for the use of these species is the manner of land management in the post-mining areas. There are many cases in which the woodlands on former PMM are subjected to intensive forest management (Podgórska 2016), or in which woodland communities of the PMM are transformed into meadow communities (Kołodziejek 2001; Holeksa et al. 2015), or even into arable fields. These anthropogenic treatments hinder the growth of the indicator species (the quantitative and qualitative share of indicator species is very low) or even make it impossible for them to occur (Strzeleczek et al. 2017). Therefore, indicator species for the PMM can occur in former post-mining habitats, where secondary succession is not significantly disturbed by anthropogenic factors and where natural forest communities have the chance to grow in harmony with soil substrate, which was created as a result of former iron mining activity. Such conditions are fulfilled on the studied former lead ore PMM in the Karczówka Nature Reserve, in which no land management is carried out and where the advanced stages of succession are observed, with the quantitative and qualitative share of indicator species.

## Conclusions

Former lead ore mining impacted the transformation of the soil layer in the studied area. The former lead PMM are habitat islands among the poorer untransformed habitats. They are characterized by higher pH, Ca, Pb, Mg, and K values in comparison with the soil of the untransformed areas. Changes in the chemical properties of the soil impacted the floristic diversity of the studied area. On the former lead PMM, a nearly twofold increase in the number of vascular species was noted in comparison to the untransformed areas surrounding the PMM. The studies conducted demonstrated that indicator species for former iron ore mining sites can also be a good indicator for remnants of former lead ore mining. These species show a strong preference for the soils of the former lead PMM, while in the untransformed areas, they did not exhibit even a single occurrence.

## Data Availability

All data supporting the findings of this study are available within the paper and its supplementary information. The complete list of vascular plant species that occur in the former lead ore mining area on the post-mining mounds and in the surroundings is provided in Table 2. All figures (Figs. 1 and 2) and tables (Tables 1–4) are original, and the present original results are included in this paper.
